# Communicating with ethnic minorities during COVID-19: An experimental test of the effect of self-, ingroup-, and intergroup-focused messages

**DOI:** 10.1016/j.heliyon.2023.e16629

**Published:** 2023-05-29

**Authors:** Neomi Frisch-Aviram, Siwar Hasan-Aslih, Eran Halperin

**Affiliations:** a University of California, Berkeley, CA, 94720, United States; b Stanford University, Stanford, CA, 94305, United States; c Hebrew University of Jerusalem, Jerusalem, Israel

## Abstract

Developing messaging to encourage minorities to adhere to health recommendations has been a complex task for governments worldwide during the COVID-19 crisis. Here, we propose and tests a new typology of messages among minorities that can be used to mobilize compliance and engagement. This typology comprises three messaging treatments emphasizing personal, ingroup, and intergroup benefits. We examine, via an experimental field study, whether there is a difference in the effect of these messages on two policy outcomes, social distancing and vaccine hesitancy, among the Arab minority living in Israel. The findings suggest that social messages, i.e., ingroup and intergroup messages, positively affect social distancing, while self-messaging harms social distancing compliance. Regarding vaccine intake, within the social messages tested, intergroup-focused messages were more effective than ingroup-focused messages for vaccination intentions only among citizens with low trust in the government. We discuss the findings in detail and propose new avenues in theory and practice to foster health policy compliance among minorities.

## Highlights

• Minorities noncompliance can arise due to a lack of trust based on past discrimination and unfavorable policies.
• A typology of messaging towrd minorities in helath sitiuation based on social identity theory is proposed, differentiating between self-, ingroup- and intergroup-based messages.
• Findings from two experimental studies are presented.
• The findings suggest that social messages have a more positive effect on health compliance than self-focused messages.
• The findings suggest that intergroup messages have a positive effect among minorities with low trust in the government.

## 1 Introduction

Fighting the COVID-19 pandemic requires the public to adhere to government health policy. Therefore, it is crucial to understand how different messages affect adherence across groups. A vast body of literature has addressed different approaches aimed toward fostering policy compliance [[1], [2], [3], [4], [5], [6], [7], [8], [9], [10], [11], [12], [13], [14], [15], [16], [17], [18]]. However, the messages developed to date target general society and may be less effective for minorities. This effect could be due to their experiences of marginalization and discrimination through past policies that might not have been appropriate for this population, thus decreasing their identification with and trust in the government and policies and negatively affecting their will to comply with future policies [19,20]. Thus, the government must recognize minorities' experiences and address the messages accordingly.

This paper contributes to the literature by examining messages targeting ethnic minorities' health policy compliance during different pandemic stages. In a pandemic, collective behavior must be changed because of the contagious nature of the infection [21]. Thus, minorities' compliance is crucial for society to overcome the pandemic. We rely on social identity theory [22,23] to introduce a new typology of messages that target adherence to health policy among minorities. According to social identity theory, people derive their social identity from the groups to which they belong. As these social identities determine emotions and behavior, this work sheds light on the challenges of policy compliance among minorities and how different messages can affect compliance behavior [24].

We suggest that when communicating with minorities, social messages need to consider their social identity and the relative standing of their group in the social system. Such a social identity approach can lead to two distinct interventions. The ingroup perspective focuses on benefits to the immediate social group of minorities, while the intergroup perspective is related to benefits stemming from minorities' relations with the majority [25]. These two group-oriented perspectives are compared to a more individualistic one, focusing on the implications of compliance on the individual.

Thus, we propose and experimentally test the influence of three messaging treatments on intentions to engage in COVID-19 prevention behaviors, one personal and two social: self-focused messages that emphasize the personal benefits of prevention [24], ingroup-focused messages that highlight the benefits of prevention for the respondents' ingroup [26,27], and intergroup-focused messages that emphasize a call from the majority group to work together and comply for the benefit of the minority and majority groups alike [26,27]. Next, in study 2, we test the moderating effect of trust in the government on the relationship between the type of message and health policy compliance intentions. In the following**,** we detail the proposed typology of messages to foster governmental compliance among minorities.

## 2 Theory

Research shows that the COVID-19 pandemic has disproportionally affected minorities; thus population demonstrates higher mortality and illness rates due to crowded living environments, a need to work in essential industries and commute using public transportation, health behaviors, and health preconditions [28]. In addition, crises can negatively impact groups already stigmatized and disadvantaged in society [29]. During the COVID-19 pandemic, prejudiced attitudes and discriminatory behaviors toward minority groups increased [30,31]. Thus, a recently published paper referred to the COVID-19 pandemic as a "syndemic" [32]. This concept describes the interactions between biological diseases and adverse social conditions, such as growing inequality and prejudice [33].

In addition to being more vulnerable, minorities are less likely to adhere to health recommendations during the COVID-19 pandemic [34]. There are two main reasons for this. First, while a shared group identity facilitates the adoption of preventative health behavior for the sake of the ingroup [35], negative experiences signal to minorities that they are not fully included as part of the national ingroup due to their racial, ethnic, and religious differences [36]. These adverse experiences may prevent minorities from identifying with the national majority group and, thus, the society in which they live [37].

Second, research demonstrates that compliance with health policy strongly correlates with feelings of belonging to the state and trust in government leadership and institutions [38,39]. For example, using a double-difference approach around the time of the lockdown announcements, Bargain and Aminjonov [2] found that high-trust regions in Europe decreased their mobility significantly more than low-trust regions. However, minorities experiencing discrimination may have less trust in government institutions and the policies they issue [40]. Thus, minority compliance demands identification with the state and a feeling of social inclusion, both of which may not be part of minorities’ life experiences.

Distrust may be even more prevalent in contexts of intergroup conflict, especially when the conflict is prolonged and characterized by a history of mutual violence, hostility, and fear [41]. In countries where a majority ethnic group heads a multiethnic society, the majority group controls politics and policies. Therefore, minorities may be denied equal rights to citizenship, immigration and civil liberties and face systematic barriers to inclusion and fair treatment in housing, education, employment, and the criminal justice system [42,43]. In this environment, minorities may be suspicious of government policies and communication, even in a seemingly 'neutral' policy arena such as health [44,45]. This mistrust may lead to poor health outcomes among minorities [45,46].

While minorities' social identities and complexities in relation to policy are well documented in the literature, and despite vast experimental research on messages used to foster policy compliance during the pandemic, most current research on policy communication during the pandemic has focused on messages targeting the general society. To fill this gap, we propose and test a typology of messages that can be used to communicate health policies and mobilize engagement and compliance. The typology builds on the classic self-focused versus other-focused distinction and adds the element of group identity to social messages to consider ingroup and intergroup perspectives.

According to social identity theory, "social groups, whether large demographic categories or small task-oriented teams, provide their members with a shared identity that prescribes and evaluates who they are, what they should believe and how they should behave" [47]. As this theory focuses on how social groups shape the attitudes and behaviors of individuals belonging to them, it is crucial for understanding their health behaviors [35]. One meaningful way social groups can shape individuals' perceptions and behaviors is by defining an outgroup for every ingroup and creating a competitive struggle between these ingroups and outgroups. Thus, we evaluate the impact of three messaging treatments on intentions to engage in COVID-19 prevention behaviors: a self-focused message, an ingroup-focused message, and an intergroup-focused message. The conceptual definitions of these messages and examples are summarized in Table 1.

**Table 1:** Typology of messages aimed toward fostering minorities’ health compliance.

Message Strategy	Conceptual Definition	Examples from Manipulation Text
**Self**	Refers to messages in which the personal benefits of prevention are emphasized	**“**We must internalize that this conduct can cost each of us dearly; it happened to me, and it can happen to you too. When it happens, our lives are in danger, but no less so - our future and dreams are in trouble. For our own sake, the chain of adhesives must be stopped; I maintain social distancing too!"
**Ingroup**	Refers to messages in which the benefits of prevention for the minority ingroup are emphasized	**“**I believe we have the power to control morbidity in Arab society. We must maintain and adapt to the new reality and find alternative ways to claim social ties and practice our tradition. Because I care about our community, I maintain social distancing too!”
**Intergroup**	Refers to messages in which the benefits of prevention for the minority and majority groups as one society are emphasized	**“**This is the first time in years that I feel that the representatives of the Israeli government care about us as a society, that they treat us as equals and that there is a chance for a real partnership. For the health and future of us all, Arabs and Jews, I maintain social distancing too!"

The present research aimed to test the effects of these three messages on a minority group in Israel. Arab citizens are an ethnic-national minority in Israel, constituting 21% of the population, including Muslims (83%), Christians (9%), and Druze (8%) [48]. Arabs in Israel experience various forms of socioeconomic disadvantages, including those related to health. For example, Arab citizens have higher infant mortality rate and shorter life expectancy than the Jewish majority [49]. Thus, similar to minorities in other countries, Arab citizens of Israel were at greater risk during the pandemic, and studies have found excess mortality rates among the Arab minority [50].

These circumstances have resulted in substantial psychological implications. A study found high levels of emotional distress and low levels of interpersonal and transpersonal hope among Arab citizens in comparison with those in Jews in Israel during COVID-19 [51]. Furthermore, in terms of adherence to government health policy, Arab citizens had lower compliance rates due to low trust in the government and religious and cultural norms [52].‏

Since the pandemic outbreak, Israel has experienced four surges in daily cases and three lockdowns to contain the spread of the disease. Nevertheless, as early as December 2020, Israel offered free vaccination to all its citizens. The effect of the messages proposed in the typology was tested on two compliance outcomes: social distancing during the beginning of the third lockdown (Study 1, Hypotheses 1 + 2) and vaccination uptake (Study 2, Hypothesis 3) during the first weeks of the vaccination rollout. In study 1, the focus is on comparing the effect of the different messages. In study 2, the moderation of trust in government is added to overcome study 1's limitation of not testing the boundary condition under which the different messages affect vaccine hesitancy.

## 3 Study 1

The goal of Study 1 was to test the effect of self-, ingroup- and intergroup-focused messages on social distancing compliance. Two research questions guided this study: a. *Among minorities, which messages have a stronger effect on health policy compliance: social-focused messages (*i.e.*, ingroup and intergroup) or self-focused message*s? b. When focusing on social messages, *among minorities, which messages have a stronger effect on health policy compliance: ingroup-focused messages or intergroup-focused messages?*

Research has demonstrated that social messages have a more significant effect on health policy compliance in general society than self-focused messages [13,15,[53], [54], [55]]. This finding could be explained by the ability of social messages to induce compassion and activate social norms, altruism, or moral duties that guide behavior [14]. Jordan et al. [24] tested three messaging treatments emphasizing the personal, public, and personal public benefits of COVID-19 prevention. They found that social messages were more effective than personal messages in increasing prevention intentions. Specifically, their work demonstrated that perceived public threat predicted prevention intentions more strongly than perceived personal threat. Likewise, Capraro and Barcelo [56] found that socially oriented messaging increased intentions to wear face masks.

However, this line of research has not considered the role of the complex social identity of minorities in shaping policy compliance. Therefore, in the typology suggested (Table 1), we propose that when targeting minority compliance, there is a need to further classify and test the effect of two types of social messages: ingroup-focused messages and intergroup-focused messages.

Pandemics may improve intergroup relations by inducing the feeling of sharing the same global traumatizing experience and uniting against a common natural enemy [20,57,58]. These effects are known as 'rally‐round‐the‐flag' effects and involve a tendency to increase support for figures or institutions associated with the general intergroup nation [59]. Because crises can reinforce the sense of a large, shared community of intersocial groups, intergroup relations may improve when groups are no longer perceived as opposed but rather as united in confronting a shared challenge [60]. Therefore, messages from the majority group that communicate positive and unifying messages toward the minority group may cause a positive reaction among minorities [61] and foster adherence to health policies [62]. However, when this effect is compared to an ingroup social message, we hypothesize that the ingroup-focused message will have a stronger effect.

According to self-categorization theory [63], people's social identities—their sense of selfhood defined by their ingroup memberships—provide them with psychologically meaningful frames to define themselves and navigate the world. Specifically, in times of crisis when the external environment becomes less stable and less predictable, people may look for comfort within their immediate ingroup, strengthening their ingroup identification. Studies have shown that traumatic experiences such as war [64], terrorism [65], or sustained intergroup conflict [66] may cause more robust identification with the immediate social group, the ingroup.

Building on self-categorization theory, previous psychological research on persuasion demonstrates that ingroup messages can be more persuasive than outgroup messages [67]. This is linked to depersonalization, the perception of similarity between the self and the ingroup. Due to this psychological process, messages from ingroup sources may be considered more credible, leading people to identify with them to a greater extent [68]. As such, a message that is linked to ingroup identification should be more persuasive than an intergroup-focused message because of the mechanism of social identity [69]. Furthermore, in the context of minorities, specifically minorities in conflictual environments, the psychological effect of ingroup identification, cohesiveness, and solidarity is more substantial as an outcome of the conflict, which widens the gap between "us" and "them" [70,71]. Taken together, we hypothesize the following.

Hypothesis 1 (H1): Among minorities, social-focused messages have a stronger effect on health policy compliance than self-focused messages.

Hypothesis 2 (H2): Among minorities, an ingroup-focused message has a stronger effect on health policy compliance than an intergroup-focused message.

## 4 Materials and method

The Institutional Ethical Review Board approved the study procedures at the Hebrew University of Jerusalem, approval number: 2021-01-281. All of the participants completed a consent form. An online experiment was conducted with four conditions (message type: control vs. self vs. ingroup vs. intergroup). The preregistration of the experiment and the Pre Analysis Plan (PAP) can be found at the OSF Registries (https://osf.io/e8yqc/).

### 4.1 Participants

The participants, adult Arab citizens, were recruited via iPanel, an Israeli internet research panel company. A power analysis determined the sample size. We aimed to detect medium changes across four condition groups (f = 0.25) with high sensitivity (95% power at the p = .01 level). A power analysis conducted in G*Power indicated that a sample of 239 was needed. A total of 314 respondents completed the survey. We filtered out observations that failed the manipulation comprehension checks, leading to a remaining sample of 240 (mean age = 29.39; 67.4% women). Most participants were of low to average socioeconomic status (SES; 45.6% low SES, 45.6% low–middle SES). A total of 50.4% had only a high school education, and 49.6% had a bachelor's degree or higher. A total of 71.7% of the sample was Muslim, 17.5% was Christian, and 9.2% was Druze.

### 4.2 Procedure

The participants answered questions regarding their past social distancing compliance to control for randomization failures. They were then randomly assigned to read one of four Facebook posts. The post featured a young Arab citizen writing a personal post explaining why he shared another post that called for people to adhere to social distancing. Four conditions were included: (i) control (neutral message, n = 60); (ii) self (social distancing for one's own sake, n = 56); (iii) ingroup (social distancing for the good of Arab society, n = 62); and (iv) intergroup (social distancing for the good of the joint society of Arabs and Jews as a request from the majority group toward the minority group, n = 62).

Following the randomized manipulation, the participants were asked to answer questions regarding their future intentions to comply with social distancing and other measures used for analysis. In addition, the participants completed a demographic questionnaire surveying their gender, age, education, income, and religion.

### 4.3 Measures

#### 4.3.1 Intentions to comply with social distancing

Social distancing intentions were measured using five items assessing the intention to comply with social distancing (adapted from Ref. [15]). These actions were “physically keep 2 m away from others,” “wear a face mask,” "wash hands frequently", "shake hands, hug, or kiss people other than members of your household (Rev.)" and “take part in religious and/or social events (Rev., e.g., weddings, funerals, parties)". The participants indicated the extent to which they were willing to participate in these actions on a 100-point scale (1 = not at all, 100 = very much so). Following an exploratory factor analysis (see Table 2), we found that these items loaded on two subscales: the preventative measures scale (distancing, masking, hand washing) and the social interaction scale (taking part in social events, shaking hands). We used the preventative measures subscale to capture the primary policy guidance from governmental bodies during the specific study (Cronbach's α = 0.73). The analysis for the second scale is shown in the supplementary material.

**Table 2:** Exploratory factor analysis with oblique rotation.

Item	F1:	F2:
**F1: Preventative Measures Scale**	Preventative Measures	Social Interaction
Physically keep 2 m away from others	.810	
1. Wear a face mask	.860	
2. Wash hands or use hand sanitizer as soon as you get home	.632	
**F2: Social Interaction Scale**		
1. Shake hands, hug or kiss people other than members of household (Rev.)		.842
2. Take part in religious and/or social events (e.g., weddings, funerals, parties)		.876

## 5 Results

No intercondition differences were found in the past preventative measures, confirming random assignment and allowing a comparison of the treatment effect.

To examine the experimental effects on the dependent variable, future intentions to take preventative measures, we ran a univariate ANCOVA with the condition (control, self, ingroup and intergroup) as a between-subjects variable and age and gender as covariates. Fisher's LSD post hoc tests were used to examine differences between the four conditions. There was a statistically significant difference in future intentions to take preventative measures between conditions [F(3, 234) = 3.98, p = .001, partial η2 = 0.049].

A pairwise comparison demonstrated that the participants in the self-focused condition reported significantly lower future intentions to take preventative measures scores than the participants in the ingroup condition (p = .001) as well as the intergroup condition (p = .011). This further indicates that social messages positively affect future intentions to take preventative measures to a greater extent than self-focused messages, supporting H1.

A comparison between the control and other conditions demonstrated a nonsignificant difference between groups that trended in the hypothesized direction. The participants in the ingroup condition displayed a nonsignificantly higher score for future intentions to take preventative measures than the control condition (p = .086). The participants in the self-focused condition displayed a nonsignificantly lower score for the future intention to take preventative measures than those in the control condition (p = .123).

When comparing the social messages, i.e., the ingroup and intergroup messages, the score for future intentions to take preventative measures in the ingroup condition was not significantly higher than in the intergroup condition. Thus, the findings do not support Hypothesis 2; the ingroup-focused message did not have a stronger effect on the future intention to take preventative measures than the intergroup-focused message. The means and SDs are reported in Table 3.

**Table 3:** Means, SE and P values of Dependent Variables by Condition (Study 1 + 2).

Variable	Mean	SE	P value-vs. control
Social distancing intentions (Study 1)			
Control	80.85	19.91	–
Self	75.08	26.72	n.s
Ingroup	87.13	19.41	n.s
Intergroup	84.59	19.70	n.s
***Intention to get vaccinated (Study 2)***			
Control	5.04	.19	–
Ingroup	5.16	.18	n.s
Intergroup	5.15	.20	n.s

In summary, Study 1 indicated that social messages (ingroup and intergroup) had a more positive effect on social distancing compliance than self-focused messages. We return to this point in the discussion section of the paper.

The limitations of this first study are as follows: first, the study failed to reveal how the two social messages (i.e., ingroup and intergroup) differ from one another and which boundary conditions exist to explain these differences (Hypothesis 2). Second, the theory section proposes trust in government as a significant barrier to minorities' policy compliance. However, this was not tested in study 1. Therefore, we conducted a second study in which we manipulated social messages and tested their effects on different policy outcomes to address these concerns.

## 6 Study 2

The justification for Study 2 is twofold. First, following the finding that social messages positively affected social distancing compliance more so than self-focused messages, Study 2 aimed to better understand the boundary conditions of the forms of social messages, ingroup and intergroup. Moreover, as past research demonstrates that vaccine intake depends on trust in the government, we focused on this boundary condition. Therefore, the guiding question for this study was as follows: *under what conditions do ingroup and intergroup messages have different effects on health policy compliance?* Next, we detail the theory behind this moderation.

COVID-19 vaccine hesitancy is defined as a postponement in reception or rejection despite vaccine availability [45]. Research has demonstrated an inverse correlation between trust in the government and COVID-19 vaccine hesitancy [72,73]. Recent studies have demonstrated that COVID-19 vaccine hesitancy is particularly high among minorities in the UK [74] and the US [75,76]. A recent review of 13 studies (n = 107,841) revealed that vaccine hesitancy was much higher among African Americans (41.6%) and Hispanics (30.2%) than among adult Americans in general (26.3%) [77].

Vaccine hesitancy among minorities is related to historical mistrust in the government and public health bodies related to vaccines that runs deep in some ethnic minority groups [45]. This mistrust is also connected to the life experiences of many minorities. Savoia et al. [78] found that experience with racial discrimination was a predictor of COVID-19 vaccine hesitancy, with a 21% higher chance of vaccine hesitancy among those reporting racial discrimination versus those who did not.

Campaigns targeting vaccine hesitancy should address the link between lower levels of trust among minorities and higher levels of vaccine hesitancy. Using the typology presented in this paper, we suggest that an intergroup-focused inclusive message that considers social mistrust and calls for partnership and reconciliation can help fight vaccine hesitancy, specifically among minorities with low trust in the government [79].‏ We therefore hypothesize the following.

Hypothesis 3 (H3): Trust in the government moderates the relationship between types of social messages and health policy compliance such that at low levels of trust in the government, an intergroup-focused message positively affects health policy compliance.

## 7 Materials and methods

Study 2 was conducted a month after Study 1 (late January 2021), when Israel began its vaccination program. In Israel, the vaccination rollout of COVID-19 vaccines started on December 20, 2020, and by the time of data collection, approximately 900,000 people had been administered the first shot. During data collection, Israel changed the vaccination allocation policy and allowed all people above 40 to get vaccinated. Although vaccination was made accessible and available, vaccination compliance remained deficient among Arab citizens, unlike among Jewish citizens (45% of the population above 60 among Arab citizens compared to 74% in the general population above 60; data obtained from Israel's Ministry of Health Dashboard by date).

### 7.1 Participants

An online experiment was conducted with three conditions (message type: control vs. ingroup vs. intergroup). The survey experiment was conducted online. The subjects, adult Arab citizens, were recruited via GeoCartography, an Israeli internet research panel company. A power analysis determined the sample size. We aimed to detect an interaction effect with medium changes across 3 condition groups (f = 0.25) with high sensitivity (95% power at the p = .01 level). A power analysis conducted in G*Power indicated that a sample of 251 was needed. A total of 306 respondents completed the survey. Of these, we filtered out people who had been vaccinated and those who failed the manipulation comprehension check, leading to a remaining sample of 237 (mean age = 32.65, 72.2% women). Most participants had a low to average socioeconomic status (SES; 42.2% low SES, 46.5% low–middle SES). This sample was more educated; 27.8% had only a high school education, and 72.2% had obtained a bachelor's degree or higher. The religious distribution was similar to that of the Study 1 sample: 72.2% of the sample was Muslim, 13.5% was Christian and 13.5% was Druze.

### 7.2 Procedure

The procedure was similar to that in Study 1. The Facebook post encouraged vaccination against COVID-19 (vs. social distancing in Study 1). Since this study focused on the effect of social messages and since data collection in Arab society is highly challenging, we omitted the self-focused condition and included only three conditions: (i) control (neutral message, n = 81); (ii) ingroup (vaccination for the good of Arab society, n = 85); and intergroup (iii) (vaccination for the good of Arabs and Jews together, n = 71). All manipulation posts are included in the supplementary materials.

Following the randomized manipulation, the participants were asked to answer questions regarding their future intentions to get vaccinated and additional measures.

### 7.3 Measures

#### 7.3.1 Intentions to get vaccinated

The intention to get vaccinated was measured using five items assessing the intention to get vaccinated (adapted from Ref. [80]). These items included "I plan to get vaccinated with the coronavirus vaccine"; "I encourage people who are particularly vulnerable to COVID-19 to get vaccinated with the coronavirus vaccine"; "I encourage close relatives to get vaccinated with the coronavirus vaccine"; "I wish all of Arab society would get vaccinated with the coronavirus vaccine"; and "I wish all of Israeli society would get vaccinated with the coronavirus vaccine". The participants indicated the extent to which they agreed with these items on a 7-point scale (1 = do not agree, 7 = very much agree) (Cronbach's α = 0.96).

#### 7.3.2 Trust in government

Trust in the government was measured using one item: "I trust the Israeli government to make sure the coronavirus vaccine is safe". The participants indicated the extent to which they agreed with this item on a 7-point scale (1 = do not agree, 7 = very much agree). This item was chosen because it was specifically related to vaccine hesitancy, and it is common in this literature to use one-item measurement to measure trust [81].

## 8 Results

Generally, the individual intention to get vaccinated *(M = 4.76; SE = 1.99)* was lower than the social intention: encouraging people who are particularly vulnerable to COVID-19 to get vaccinated with the coronavirus vaccine *(M = 5.31; SE = 1.75)*; encouraging close relatives to get vaccinated with the coronavirus vaccine *(M = 5.03; SE = 1.78)*; wishing that all of Arab society would get vaccinated with the coronavirus vaccine *(M = 5.14; SE = 1.79)*; and wishing that all of Israeli society would get vaccinated with the coronavirus vaccine *(M = 5.25; SE = 1.7)*.

To examine the effects of the three conditions on future intentions to get vaccinated, we ran an ANOVA with the condition (control vs. ingroup vs. intergroup) as a between-subject variable. We did not control for demographic variables because they were not significantly correlated with the variables and did not differ across the conditions. There was no significant main effect of condition on future intentions to get vaccinated. The ingroup- and intergroup-focused messages were not significantly different from the control message *(F(2, 234) = 0.133, p = .876*) or each other *(t(160) = 0.55, p = n.s).*

To test whether the effect of messages depends on trust levels, we ran a moderation analysis using Hayes’ [82] PROCESS bootstrapping macro (Model 4; 5000 iterations). The model was specified with the condition as the independent variable, trust in the government as the moderator variable and the intention to get vaccinated as the outcome variable.

**Fig. 1:**
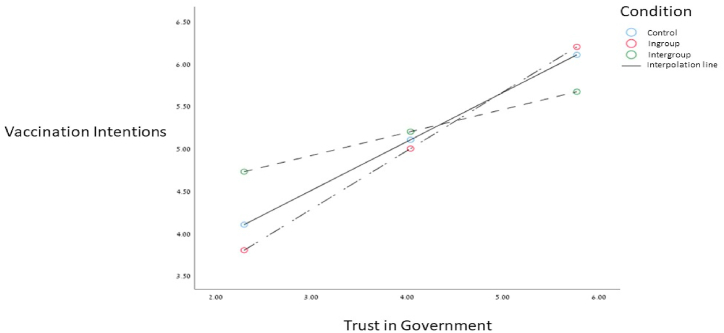
Moderation of trust in government on the relationship between the condition and vaccine intention.

The full model accounted for 31.7% of the variance in the intention to get vaccinated (R2 = 0.317, F(5, 231) = 21.46, p < .001). Because the independent variable was categorical, two interactions were generated, with only the interaction of the second category (trust*intergroup-focused message) demonstrating significance (*b = -0.305, p*<.0*5; 95% [LLCI = -0.572, ULCI = -0.038]*). The intergroup-focused messages significantly affected those with low trust (*b = .622, p = .07; 95% [LLCI = -0.073, ULCI = 1.31]*) and did not affect those with high trust. Table 4 lists all slopes, plotting the interaction in Fig. 1.

**Table 4:** Moderation of trust in government on the relationship between the condition and vaccination intention (H3).

	Dependent Variable = Vaccination Intentions R2 = .317***	
	B	T
Constant	2.865	7.674***
Ingroup-focused message	−.552	−1.006
Intergroup-focused message	1.234	2.11*
Trust in government	.566	6.44***
Ingroup X trust in government	.102	.824
Intergroup X trust in government	−.305	−2.255**
Ingroup X trust in government	Simple Slopes for Trust in Government	
	Effect	T
-1SD (Low trust in government)	−.348	−1.029
Mean (Medium trust in government)	−.144	−.644
+1SD (High trust in government)	.059	.181
Intergroup X trust in government		
-1SD (Low trust in government)	.622	1.762*
Mean (Medium trust in government)	.011	.047
+1SD (High trust in government)	−.600	.098

N = 237; *p < .08, **p < .01, ***p < .001

This finding may further explain why we did not observe a main effect. The intergroup-focused message had a more positive effect than the control message only among those with low trust in the government. There were no direct or moderating effects of the ingroup-focused message on the intention to get vaccinated. However, the intergroup-focused message was effective specifically for those most in need, i.e., those low in trust.

## 9 Discussion and conclusions

This research aimed to expand current knowledge on the effect of messages that foster adherence to health policy guidelines among minorities. Theoretically, we acknowledge the unique challenges of policy compliance among minorities and proposes a new typology of messages to foster policy compliance among minorities. This typology is based on social identity theory [22] and recognizes that policy compliance among minorities may be complex due to past discrimination and policies that might have harmed them. Thus, the government must communicate better during health crises. This paper focuses on how to improve this communication.

Next, this paper experimentally tested the effect of the three forms of messages in the suggested typology, i.e., self, ingroup, and intergroup, on two policy compliance outcomes: social distancing (Study 1) and intentions to get vaccinated (Study 2). Specifically, the experiments aimed to answer the following research questions: *what is the effect of social-focused messages (*i.e.*, ingroup and intergroup)* vs*. self-focused messages on minority health policy compliance; what is the effect of ingroup-focused messages* vs*. intergroup-focused messages on minority health policy compliance; and under what conditions do ingroup and intergroup messages have different effects on health policy compliance?*

Regarding the first question, consistent with recent experimental research [13,15,53,54] and supporting Hypothesis 1, the current study demonstrates that social messages positively affect health guideline adherence to a greater extent than self-focused messages among minorities. This effect is possibly explained in past research that found that minorities develop stronger social ties with their ingroup to reduce self-uncertainty [83] and overcome prejudice threats [84]. This effect explains why social messages that appeal to a sense of social belonging and inclusion are more salient than self-focused messages, specifically among minorities.

Regarding the second question, while past studies point to the importance of ingroup identity for minorities as opposed to intergroup connectedness [69], the findings do not demonstrate that ingroup-focused messages induce stronger compliance than intergroup-focused messages in both studies (a main effect), as suggested in Hypothesis 2. This finding may be related to a ceiling effect because most values obtained for the dependent variables in both studies approached the upper limit of the scale, as commonly observed in online samples. Thus, the findings do not fully answer the second research question.

Regarding the third question, the findings demonstrate that intergroup-focused messages can be more effective in certain situations than ingroup-focused messages for certain people, specifically minorities with low trust in the government, supporting Hypothesis 3. This finding is consistent with the theoretical approach that views adherence to policy and guidelines as strongly related to trust in the government and aligns with similar findings during the pandemic, which found a strong correlation between trust in the government and compliance with health guidelines [20,38,39].

Trust is based on the sense of legitimacy of the government and policy, the people and processes [[85], [86], [87]]. As such, when trust in the government is low, trust in guidelines and policies might decrease. The findings emphasize this link and point to the importance of conveying different messages to people with low trust in the government. The higher vaccine hesitancy among minorities indicates that minorities with low trust in the government may be receptive to information campaigns that draw on intergroup messaging that emphasizes a call from the majority group to work together and adhere to guidelines for the benefit of the minority and majority groups alike, which may positively affect compliance. Minorities’ lack of trust might be mitigated by using shared goal–focused messages.

The paper's strengths are, first, its unique focus on ethnic minorities' challenges with health policy compliance. While much has been written about health policy compliance during the pandemic, and despite empirical evidence on the fatal outcomes of minorities' noncompliance worldwide, few studies have focused on the causes of noncompliance among minorities. Second, this study goes a step further and proposes a typology of messages that can foster compliance among ethnic minorities based on social identity theory. This typology can form the basis for future research. Third, we used an experimental research design to test the typology and answered three questions. The findings indicate that social messages, i.e., ingroup and intergroup messages, positively affect social distancing, while self-focused messaging harms social distancing compliance. Regarding vaccine intake, within the social messages tested, intergroup-focused messages were more effective than ingroup-focused messages for vaccination intentions only among citizens with low trust in the government.

The studies reported here have some limitations and weaknesses that must be highlighted. First, concerning the internal validity of the studies, the measures might entail social desirability bias because they are self-reported measures, which might explain the reported high mean of compliance. However, following Daoust et al. [88], we maintain that this does not alter the inferential conclusions because the bias is homogenous across the study subgroups. Moreover, limitations of measuring intentions rather than actual behavior must be considered. While experimental evidence demonstrates that changes often follow behavioral intentions in behavior, we cannot rule out the possibility that an intention‒behavior gap exists and that the reported intentions do not reflect actual behavior [89].

Second, the nature of the Israeli–Palestinian context may limit the generalizability of the findings to other social contexts. Because this is a case of an intractable ethnic conflict that includes violent actions, it is possible that the challenge of policy compliance among ethnic minorities is greater than in many other societies. Notably, we speculate that the distinctions between the social messages, ingroup-vs. intergroup-focused messages, could be less dominant in a less conflictual environment. Future work should test the suggested typology in different intergroup settings, such as across racial and religious divides and in different societies.

Researchers could use the typology suggested in this paper and the initial findings for further empirical examination. For example, exploring how policy communication through the types of messages in the proposed typology affects compliance in different policy domains, such as environmental policy compliance or tax returns. While we aim to theoretically explain why certain messages may be more effective in fostering adherence to health guidelines among minorities than others by discussing mediators such as enhanced societal belonging or increased trust in societal institutions, we did not test these mediators experimentally. Future research should include some of the theoretical foundations in this paper to expand knowledge of messaging in relation to minorities.

The study findings have practical implications. Although previous studies have examined different messages aimed toward fostering compliance during the COVID-19 pandemic, the unique aspects of minorities' emotions and behaviors in such a context have been neglected. Following Weaver [18], this work suggests that a three-step process should be used to encourage minorities to comply: first, acknowledging the heterogeneity within the target population and specifically the challenge of policy compliance among minorities; second, to understand barriers that may keep individuals from complying with health policies, specifically minorities who might have experienced past discrimination and harmful policies; third, matching the campaigns so that they address the most important barriers that inhibit compliance. As demonstrated here intergroup messages can be more effective among minorities with low trust in government. By bringing together the literature on intergroup relations with work on policy compliance [90,91], this work contributes to understanding how different messages affect minority policy compliance during pandemics. Thus, the current work extends past research on policy compliance by examining how a minority social group perceives messages based on past experiences and how such perception affects behavioral outcomes.

In summary, the findings underscore the importance of (a) considering minorities as a unique target audience for governmental messages and (b) considering social messages rather than self-focused messages and, more specifically, intergroup-focused messages when there is low trust in the government among minorities. As it is the states' responsibility to become more trustworthy and accessible toward minorities, government officials can use the typology and findings of this paper when planning campaigns.

## Author contribution statement

Neomi Frisch Aviram; Siwar Hasan-Aslih; Eran Halperin: Conceived and designed the experiments; Performed the experiments; Analyzed and interpreted the data; Contributed reagents, materials, analysis tools or data; Wrote the paper.

## Data availability statement

Data will be made available on request.

## Declaration of competing interest

The authors declare that they have no known competing financial interests or personal relationships that could have appeared to influence the work reported in this paper
