# Supplementary material for: Communicating with ethnic minorities during COVID-19: An experimental test of the effect of self-, ingroup-, and intergroup-focused messages
Source: Heliyon. 2023 May 29;9(6):e16629. doi: 10.1016/j.heliyon.2023.e16629 (PMC10226279; doi:10.1016/j.heliyon.2023.e16629)
Supplement: Multimedia component 1 [file mmc1.docx]

**Appendix: Manipulation Texts (translated from Arabic)**

**Social Distancing (Study 1)**

The manipulation was designed as four Facebook posts. The post included a young Arab citizen who wrote a personal post explaining why he shared another post that calls to adhere to social distancing.

**CONTROL**


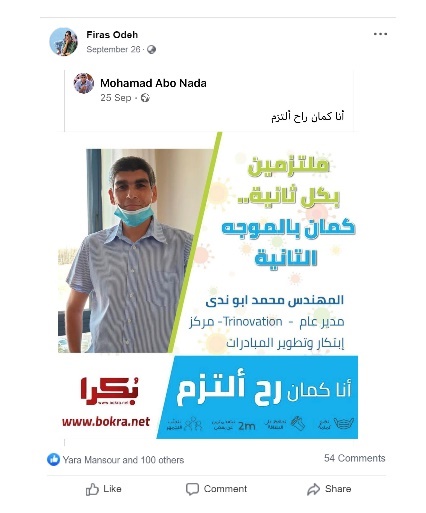


**SELF**


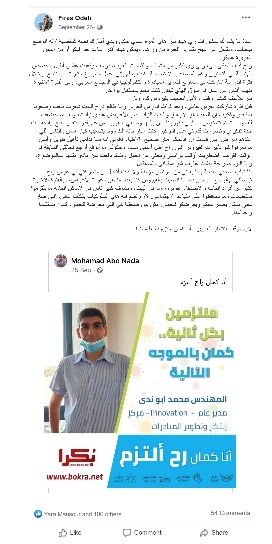
I feel that this time I must share as the situation is urgent. I want to share a personal story in the hope that I will be able to get the message across and maybe help one of you or your loved ones.

I will start by saying that like many Arab young men and women, I studied and worked very hard to be accepted to study engineering at the Technion despite the economic difficulties. My dream is to set up a successful start-up. During my studies, I took part in projects to promote entrepreneurship and technology in the Arab society, and recently I started looking for a job at one of the high-tech companies. The future really looked promising!

But everything stopped .. because I got sick in the Coronavirus.

Three weeks ago, I spent time at a family wedding in the village. Three days later when I went up the stairs, I felt tired and had difficulty breathing but I thought to myself it was due to overexertion. After the symptoms worsened, I tested myself and was diagnosed with the Coronavirus. Since then, my condition has been deteriorating. I cannot breathe well, my thinking ability has decreased and I have impaired ability to concentrate. Contrary to what I thought before (that Coronavirus only hurts adults, etc.), the doctors tell me that I have another long rehabilitation and they do not yet know what effects will stay with me for a while .. In the meantime, I stopped my studies and my job search. As a result I lost a lot of money and I'm really not sure about the future anymore...

I am a healthy guy without any background illnesses, and I did not imagine that a visit to a family wedding would end this way… We receive invitations to events every day, people come to visit us at home, friends and family invite me to spend some time with them, and in the public sphere I see many people who do not keep social distancing. We must internalize that this conduct can cost us dearly, it happened to me and it can happen to you too. When that happens, our lives are in danger, but no less so - our future and dreams are in danger.

For our own sake, the chain of adhesives must be stopped, I keep social distancing too!

**INGROUP**


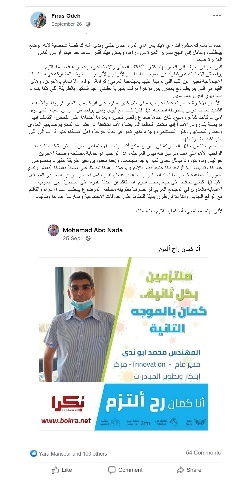


I feel that this time I must share, the situation really requires it. I want to share a personal story in the hope that I will be able to convey the message and perhaps help any of you or your loved ones.

Anyone who knows me knows that I am a person for whom family and social cohesion is very important and that is why I make sure to fulfill my social duties and participate in all the social, unfortunate and fortunate events. Participating in social events for me is an expression of important values ​​we grew up with in Arab society: respect, loyalty and concern for others that connect us together. But I recently experienced something that made me rethink how we apply our values.

I have recently attended several weddings despite the Coronavirus warnings, as many of you I guess. Unfortunately, I learned the hard way. Two weeks ago I spent time with my parents at a family wedding, and the next day my mother did not feel well. She suffered from headaches and shortness of breath, and we took her for tests. It turned out that she was sick with Coronavirus. And what's worse is that she was not the only one, we heard that dozens of other people were infected in that wedding, some of whom were hospitalized, and some in critical condition. Parents of my close friends who are not sure they will ever overcome it.

We may have fulfilled our social duty to attend events, but I'm not sure we have fulfilled the deeper duty we have now and that is to take care of others around us. The coronavirus poses a significant challenge for us as a society and it forces us to think differently about our values. If we really care about each other, we should also maintain each other's health and show more group responsibility, for all of us and especially for the older and weaker among us. For us to continue to be in each other's happy events in the future, we must first take care of each other today. I believe we have the power to control the morbidity in the Arab society if we act differently, it will require us to maintain and adapt to the new reality and find alternative ways to maintain social ties and practice our tradition. Because I care about our society, I keep social distancing too!

**INTERGROUP**


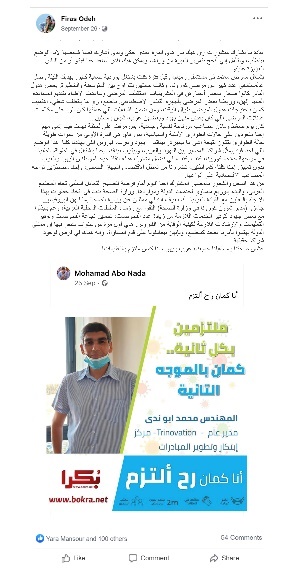
I feel that this time I have to share, as the situation really requires it. I want to share something a bit personal in the hope that I will be able to convey the message.

I am a certified nurse at Rambam Hospital. Two weeks ago I had a very difficult shift, a number of Coronavirus patients came to us in moderate to severe condition, some were really young in their 20s. I accepted the patients and helped the doctors in providing medical assistance, and we connected some of them to artificial respiration. As part of my job, I took care of their needs all the time, including answering the families of the patients on the phone. These patients were Jews and Arabs, young and old ..

It was a very busy day physically and mentally, but I had a significant moment when I realized something ... We are used to security and political emergencies, and this is the first time in many years that the emergency comes from a factor that does not distinguish between us - Jews and Arabs. A factor that illustrates the shared destiny between Jews and Arabs and makes us close to each other. In the Corona epidemic, us, the medical teams are working from together in a joint struggle to save the lives of both Jewish and Arab citizens. All of us as citizens were also harmed as a result of the shutdown of the economy and education systems and we must deal with various economic difficulties.

From this sense of partnership and joint destiny, we all have an opportunity to correct the negative attitude towards Arab society and begin with an equal distribution of state services and resources. The Ministry of Health has begun taking steps in this direction in cooperation with the Arab leadership. Representatives from the Ministry of Health, including Professor Gamzo, the Coronavirus projector, have met with heads of local authorities in Arab society, and they are making efforts to find isolation solutions, increase the number of tests and availability for epidemiological investigations and streamline testing processes. This is the first time in years that I feel that the representatives of the Israeli government really care about us as a society, that they treat us as equals and that there is a chance for a real partnership.

For the health and future of us all, Arabs and Jews, I keep social distancing too!

**Vaccination Intention (Study 2)**

**CONTROL**

As a hospital nurse, taking care of patients with Covid-19 daily, I feel that I must share my thoughts now that a vaccine against Covid-19 is available and holds real hope of taking control of the pandemic and protecting the Arab population.

I understand the dilemma over vaccination but think that even if vaccination can have side effects, it's not as dangerous as contracting Covid-19. We all want to get back to our normal life and routine, get back to meeting people, hang out and travel. Vaccination has opened the door for a real change as it can prevent infection and prevent serious illness and death.

It's time to move on, I took the vaccine!

**INGROUP**

As a hospital nurse, taking care of patients with Covid-19 daily, I feel that I must share my thoughts now that a vaccine against Covid-19 is available and holds real hope of taking control of the pandemic and protecting the Arab population.

I am a certified nurse at a big Hospital. In the last few months since the outbreak of Covid-19 my work has been much more intense. I have seen a lot of suffering and pain treating Covid-19 patients, a quite a few of whom were from the Arab community.

In this post I am writing not as a nurse, but as a person who grew up in the Arab community. Anyone who knows me knows that I believe in the values ​​of respect, loyalty and concern for others around me. Family and social cohesion is very important to me and I like to visit relatives, hang out with friends, attend joyous events and enjoy holiday visits.

This is why it's so hard for me that we cannot sustain these simple things because of Covid-19. The corona has moved people away from each other, and especially family members. We constantly live in concern for our lives and the lives of our loved ones. Unfortunately, I knew several people who were close to me and caught the virus, some who became seriously ill and some even died. It really hurts me that I could not visit them and support them during their difficult hours. To me, this is a significant part of being part of the Arab community. We all want to get back to our normal life and routine, get back to meeting people, hang out and travel. Not needing to see so much pain and suffering.

Lately, vaccination has opened the door for a real change as it can prevent infection and prevent serious illness and death, I understand the dilemma over vaccination but think that even if vaccination can have side effects, it's not as dangerous as contracting Covid-19

Covid-19 risks our lives, but it also risks our social cohesion and the values ​​we grew up on. If we really care about each other, and if we really care about our community, our parents and grandparents, we need to show more group responsibility. That’s why I think we all need to get vaccinated and convince others to get vaccinated.

Because I care about my community, I took the vaccine!

**INTERGROUP**

As a hospital nurse, taking care of patients with Covid-19 daily, I feel that I must share my thoughts now that a vaccine against Covid-19 is available and holds real hope of taking control of the pandemic and protecting the Arab population.

I am a certified nurse at a big Hospital. In the last few months since the outbreak of Covid-19 my work has been much more intense. I have seen a lot of suffering and pain treating Covid-19 patients, people in all colors and race.

It has been a very busy period physically and mentally for me, but during this period I realized something ... the Covid-19 crisis comes from a health factor that does not distinguish between people based on their race. This illustrates the shared humanity of all people from all colors and races. During the pandemic, medical teams are working in a joint struggle to save the lives of all people. All of us as citizens have been harmed as a result of the shutdown of the economy and education systems and all of us need to deal with economic difficulties. This pandemic has changed the life of all of us, Arabs and Jews alike, and we all pray to get back to our blessed routine. We all want to get back to our normal life and routine, get back to meeting people, hang out and travel. Not needing to see so much pain and suffering.

Lately, vaccination has opened the door for a real change as it can prevent infection and prevent serious illness and death, I understand the dilemma over vaccination but think that even if vaccination can have side effects, it's not as dangerous as contracting Covid-19.

Out of a sense of shared destiny and a common struggle against the pandemic there is now an opportunity to correct the negative attitude towards the Arab community and begin with an equal distribution of state services and resources. Health authorities are trying to bring vaccines to our neighborhoods as quickly and efficiently as possible. Vaccine stations are opening daily in our communities, and the state seems to be making a big effort to promote this. This is the first time in years that I feel that the representatives of the health authorities really care about us as a community, that they treat us as equals and that there is a chance for a real partnership. The more we will show a will to get vaccinated, the more we will prove that we are better partners, and we will reduce the tensions between Arabs and Jews in Israel.

Because I care about our country, I took the vaccine!

**List of Surveyed Additional Variables**

Social distancing behavior, Past vaccination behavior, Past sickness with Covid-19, Vaccination accessibility, General knowledge on Covid-19, Trust in government, Trust in healthcare system, Trust in pharmaceutical companies, Wariness about the vaccine, Skepticism and Conspiracy beliefs (vaccination and Covid-19 related); Identification and Connectedness, Group efficacy, Identification with the state, Superordinate goals/identity and Trust in government.

**Additional Analysis**

| **Variable** | **Mean** | **SD** | **P value-against control** |
| --- | --- | --- | --- |
| *Social Interaction Scale (study 1)* | | | |
| Control | 76.2 | 28.79 | -- |
| Self | 69.89 | 31.77 | n.s |
| Ingroup | 79.75 | 29.67 | n.s |
| Outgroup | 72.41 | 30.92 | n.s |

*Means, SD and P values of Dependent Variables by Condition for Social Interaction Scale*
